# Economic shocks and mental health in Bangladesh

**DOI:** 10.1136/bmjgh-2025-020502

**Published:** 2025-12-25

**Authors:** Shamma Adeeb Alam, Wameq Azfar Raza, Claus C Pörtner, Syed Shabab Wahid, Malabika Sarker

**Affiliations:** 1 Dickinson College, Carlisle, Pennsylvania, USA; 2 World Bank, Dhaka, Bangladesh; 3 Department of Economics, Seattle University, Seattle, Washington, USA; 4 Center for Studies in Demography & Ecology, University of Washington, Seattle, Washington, USA; 5 Georgetown University, Washington, District of Columbia, USA; 6 Behavioral and Social Sciences, Brown University, Providence, Rhode Island, USA; 7 Heidelberg Institute for Global Health, Heidelberg University, Heidelberg, Baden Wurttemberg, Germany

**Keywords:** Mental Health & Psychiatry, Health economics

## Abstract

**Introduction:**

Economic shocks, that is, events that cause a sudden loss of income for households, are common in low-income and middle-income countries (LMICs), yet their impact on mental health remains understudied. While such shocks may contribute to depression and anxiety, evidence remains limited, particularly in resource-poor settings where mental health disorders are underdiagnosed and undertreated. This study examines the causal impact of economic shocks on depression and anxiety in a low-income context.

**Methods:**

We used nationally representative panel data over two waves from Bangladesh, applying individual fixed effects to estimate the impact of economic shocks on mental health outcomes. Economic shocks were defined as adverse events negatively affecting income, assets or production. Depression and anxiety were assessed using the validated Patient Health Questionnaire-9 and Generalised Anxiety Disorder-7 scales.

**Results:**

In a two-round sample of 7090 observations, 16.3% (N=1155) experienced depression, 6.5% (N=459) experienced anxiety and 5.1% (N=361) experienced both depression and anxiety. Economic shocks significantly increased depression and anxiety. Individuals experiencing multiple types of shocks had nearly double the risk of reporting either mental health disorder compared with those facing a single shock. These impacts persisted for 6–12 months postshock. Adverse mental health effects were concentrated among individuals without coping mechanisms, such as use of savings, credit or support from friends and family, while those with access to such mechanisms showed no significant adverse outcomes. Education appeared protective, with individuals who had at least primary education exhibiting lower vulnerability to mental health issues in the face of shocks.

**Conclusions:**

Economic shocks have a substantial and lasting mental health impact, with compounding effects for those experiencing multiple shocks. Education and coping mechanisms may serve as a buffer against adverse outcomes. These findings highlight the need for targeted interventions to mitigate the mental health consequences of economic disruptions in LMICs.

**WHAT IS ALREADY KNOWN ON THIS TOPIC**

- Prior research from high-income countries links economic downturns to worsening mental health. In low-income and middle-income countries (LMICs), evidence is sparse and largely based on cross-sectional data and singular large-scale events, such as earthquakes. As a result, little is known about the causal impact of routine household-level economic shocks on mental health, or whether different types of shocks have different effects in LMIC contexts.

**WHAT THIS STUDY ADDS**

- This is the first study in an LMIC context to assess the mental health effects of a broad range of household-level economic shocks—including income, asset and production losses—as well as the cumulative impact of experiencing multiple shocks. Using nationally representative panel data from Bangladesh and individual fixed effects models, it provides robust causal evidence on the relationship between economic shocks and symptoms of depression and anxiety. This study highlights the importance of education and coping mechanisms, such as use of savings, credit or receiving help from friends and family, in moderating the effect on mental health following shocks.

**HOW THIS STUDY MIGHT AFFECT RESEARCH, PRACTICE OR POLICY**

- Our findings highlight the mental health burden associated with common economic shocks in LMICs, suggesting that economic vulnerability can have lasting psychological consequences. These results indicate the need to integrate mental health services into social protection and economic recovery programmes. Policies that recognise and address the mental health dimensions of poverty and income loss could improve overall public health and resilience in resource-constrained settings.

## Introduction

 Economic shocks, that is, events that cause a sudden loss of income for households, are pervasive for people in low-income and middle-income countries (LMICs), where formal insurance and social protection mechanisms are often limited. These shocks have been shown to negatively affect livelihoods, food security, child well-being and physical health.^1^,^5^ However, their consequences for mental health remain less well understood.

Addressing mental health is a global priority, outlined in Sustainable Development Goal 3.4, yet people in LMICs face severe access constraints. The treatment gap, that is, the proportion of people who have a mental health disorder but do not receive appropriate or any treatment, is as high as 85%, compared with 40% in high-income countries, exacerbating the long-term consequences of psychological distress.^6^,^8^

Given the frequency of economic shocks in the LMIC context, understanding their role in shaping mental health outcomes is crucial. While economic shocks are expected to affect mental well-being, few studies have linked them to depression or anxiety. Most of the prior research on shocks in LMICs has explored the mental health impacts of major natural disasters, such as earthquakes, and found elevated rates of depression and post-traumatic stress disorder due to widespread death and destruction.^9^,^13^ However, one study from Ecuador found a decline in mental health disorders following an earthquake, which was attributed to strengthened social cohesion.^14^

Recently, studies on LMICs have begun to explore how more common economic disruptions—such as droughts, income shocks or crop failure—may affect mental health. Research in India has linked droughts to increased rates of farmer suicides^15^ and economic shocks to higher rates of depression.^16^ Additional studies have shown that climate variability, such as extreme temperatures or excessive rainfall, can adversely affect mental health.^17^,^20^ A study across 19 African countries further demonstrated that in utero exposure to environmental shocks led to increased symptoms of mental illness in adulthood, likely due to disrupted fetal development and enduring economic hardship.^21^ More recently, studies have documented the mental health consequences of disruptions during the COVID-19 pandemic.^22^ However, since the pandemic affected nearly all individuals across multiple aspects of life, it is challenging to disentangle the mental health effects of economic losses from broader societal stressors.

Longitudinal studies, where the researcher can track individuals over time to observe how their mental health changes in response to shocks and use individual fixed effects to address the potential endogeneity of shocks and mental disorders, are rare in this literature. Outside of the context of the pandemic, only two studies used longitudinal data with individual fixed effects to examine the effect of economic shocks in LMICs. The findings are mixed. In Malawi, a study focusing on adults aged 45 and above revealed that shocks exacerbated depression and anxiety.^23^ Another study in South Africa found that the loss of property did not affect mental health, but the death of household members was associated with poor mental health.^24^

This study addresses a critical gap in the literature by examining the relationship between economic shocks and mental health—specifically symptoms of depression and anxiety—among adults of all ages in an LMIC setting. Using a longitudinal, nationally representative, household survey dataset from Bangladesh, we exploit individual fixed effects to isolate the impact of economic shocks over time, thereby strengthening causal inference. Unlike prior research that often focuses on specific subpopulations or singular types of shocks, our study examines a broad spectrum of household-level economic shocks, including income loss, asset loss and production disruptions. This wider lens allows us to examine the varied pathways through which economic losses may impact mental health. By combining validated mental health screening tools with robust empirical methods, our analysis offers a more complete understanding of how common and recurrent economic shocks can influence mental health disorders.

## Methods

### Study design and participants

We use longitudinal household-level data from Bangladesh, collected by Data International for the World Bank.^25^ The two-round survey is nationally and subnationally representative, covering urban and rural areas. The sampling method employed a two-stage, stratified sampling approach with probability proportional to size. In the first stage, 150 enumeration areas (primary sampling unit (PSU)) were selected across three strata: 90 PSUs from rural areas, 24 from the Dhaka and Chattogram city corporations, and 36 from other urban and periurban areas. Urban areas were defined as city corporations, while periurban areas encompassed other remaining urbanised regions.

In the second stage, a household listing exercise was conducted in each PSU to identify eligible households based on specific criteria: households with women and children under 5 years of age, households with older adults (aged 66 years or above), and households with other demographic combinations. Eligible households were randomly selected within each PSU using a ratio of 16:2:2 (rural areas: Dhaka and Chattogram city corporations: other urban and periurban areas), which mirrors the population distribution from the 2011 national census of Bangladesh.^25^

Survey respondents were household heads or their spouses (≥15 years). The first survey round, conducted between August and September 2019, included 3606 individuals and took place immediately after the peak of summer, characterised by high temperatures. The second round occurred during the cooler winter months in January and February 2020. The first round followed the lean season from mid-March to mid-May, while the second round was conducted after the more severe lean season, known as *monga*, from mid-September to November.^26^ Capturing data soon after both lean seasons is particularly relevant for our study, as mental health has been shown to vary with agricultural cycles and the associated periods of food and income insecurity.^27^

On the completion of the first survey round, households received mobile phone talk-time worth approximately US$2.17 (based on 2019 exchange rates) to incentivise participation. Enumerators maintained monthly contact with households to encourage retention and provided an additional US$2.17 every 2 months to those who remained engaged. The attrition rate for the second round of the survey was 3.5%.

### Mental health measures

Depression was assessed using the Patient Health Questionnaire-9 (PHQ-9), a widely used screening tool that consists of nine questions. Anxiety was assessed using the Generalised Anxiety Disorder-7 (GAD-7), a seven-question instrument. Both instruments use a 4-point Likert scale and a 2-week recall period. These instruments have been culturally adapted for use in Bangladesh and are widely employed in the country.^28^,^31^

A clinical cut-off score of 10 was used for both the PHQ-9 and GAD-7 to determine the presence of depression and anxiety. Individuals with scores of 10 or higher on either scale were considered to have depression or anxiety, respectively. This cut-off is based on established best practices,^32 33^ previous research in Bangladesh^20^ and validations from other South Asian countries.^34^ Studies have shown that the specificity and sensitivity of both measures are optimised at this threshold.^35 36^

In LMICs, where mental health professionals are scarce and few individuals seek professional help, clinical diagnoses of mental health conditions are relatively rare. Thus, the PHQ-9 and GAD-7 are especially valuable for understanding mental health challenges in these contexts. The use of the PHQ-9 to define depression has also been endorsed by the *Lancet–World Psychiatric Association Commission on Depression*.^37^ For our analysis, we created two binary variables using the threshold score of 10 to represent depression and anxiety, with a value of 1 indicating the presence of the condition and 0 indicating its absence. Additionally, we created another binary variable to capture the co-occurrence of both depression and anxiety.

### Economic shocks

Households were asked whether they had been adversely affected by any of the following economic shocks in the past 6 months: drought or irregular rainfall, floods, extreme heat events, landslides or erosion, high levels of crop pests or livestock disease, spikes in agricultural input prices, or drops in agricultural output prices. For each reported shock, respondents were asked when it first occurred and how long it lasted. Shocks involving serious illness, accidents or the death of an income earner or household member were excluded from the analysis due to their direct effects on mental health.

A binary variable was created to indicate whether a household experienced any non-health-related economic shock (1=experienced any shock, 0=did not). If a household reported a shock, they were further asked whether the shock resulted in a decline in (1) income, (2) assets or (3) food production. Based on this, three additional binary variables were constructed: income loss, asset loss and production loss, each coded as 1 if a household reported a non-health shock causing a reduction in the respective category and 0 otherwise. These variables allow for disaggregated analysis of economic impacts.

It is possible that exposure to multiple types of shocks may have stronger adverse effects on mental health. To examine this, we categorise individuals based on whether they experienced a single type of shock (such as drought, flood, extreme heat events) that led to losses in income, assets or production, or two or more types of shocks.

### Other data

The survey also collected information on the types and number of assets owned by the household. A principal component analysis was conducted to generate a composite measure of wealth. (The survey collected information on ownership and quantity of a wide range of assets. Using the reported number owned for each asset, we constructed the wealth indicator as the first principal component of asset ownership. The assets included are radio, television (TV), cable connection for TV, DVD/CD/Blu-ray player, mobile (non-smart) phone, smartphone, land phone, computer/laptop, Wi-Fi (home connection), refrigerator, wardrobe, electric fan, air conditioner, water pump, Integrated Power Supply unit (IPS)/generator, car, tempo/CNG, rickshaw/van, bicycle, motorcycle, bank account, buffaloes, cows, goat/sheep, chickens/ducks, other farm animals, amount of homestead land, amount of other land owned).

A p value of less than 0.1 was considered to indicate statistical significance for all analyses. All analyses were conducted with Stata (V.18).

### Role of the funding source

The funder of the study had no role in study design, data collection, data analysis, data interpretation or writing of the manuscript. The corresponding author had full access to all the data in the study and had final responsibility for the decision to submit for publication.

### Data availability statement

Reasonable request for anonymized data can be made to the World Bank, the institution that collected the data. Requests for access should include a clearly articulated plan of use for the third party use of data.

### Statistical analysis

We employ a linear model, ie, Ordinary Least Squares, with individual fixed effects. Using the equation below, our main specification regresses outcomes, Y, on a set of variables.

*Y_i,j,t_ = β_0_ + β_1_ Shocks_j,t_ + β_2_ X_i,j,t_ + δ_i_ + ε_i,j,t_*, (1)

where *i* denotes individual, j denotes a region, and *t* survey rounds.

The individual fixed effects, *δ_i_*, control for unobserved individual-level and household-level time-invariant factors that could bias the results. This approach allows us to control for all time-invariant characteristics associated with the individual/household, such as date of birth, gender, religion, ethnicity, constant preferences, household characteristics and area-level factors. (This means that any variable that does not change over time, and which is likely to influence our outcome variables, is captured by the individual fixed effect and would consequently drop out of the estimations).

The vector *X_i,j,t_* controls for time-varying confounders. This includes a survey round dummy to capture mental health changes over time and any seasonality in shocks. Wealth is controlled using the first principal component of household asset ownership. Finally, ε represents the error term. The standard errors are clustered at the level of PSU.

### Patient and public involvement

It was not appropriate or possible to involve patients or the public in the design, conduct, reporting or dissemination plans of our research.

## Results

3610 individuals were interviewed at baseline. Of those, 3480 remained at the follow-up round. As shown in online supplemental appendix table A1, 59.1% of respondents were female, 86.0% were married, 56.6% had completed primary education or higher, and 60.6% resided in rural areas. Men, individuals without primary education, poorer respondents and rural residents were more likely to experience shocks that resulted in income, asset or production losses than women, the more educated, wealthier individuals and urban residents. Although these differences are significant, the use of individual fixed effects accounts for all time-invariant characteristics, ensuring that these baseline differences do not bias the estimates.

Online supplemental appendix table A2 shows that in round 1 the most frequently reported shock was extreme heat events (60.6%), consistent with the survey being conducted immediately after peak summer, followed by drought or irregular rainfall (33.5%), floods (27.0%), and decreases in agricultural output prices (19.2%). In round 2, the most reported shocks were extreme heat events (27.4%), floods (20.2%), drought or irregular rainfall (18.2%), and livestock diseases (15.7%). (Two major cyclones occurred during the reference period: Cyclone Fani in May 2019 and the more destructive Cyclone Bulbul in November 2019. In our sample, 1.1% of respondents reported a natural disaster in round 1 and 5.7% in round 2. Focusing on coastal divisions, which were most affected, the share was 1.1% in round 1 and 13.4% in round 2. Although these cyclones had severe localised effects, only about 3% of households in the nationally representative sample reported a natural disaster across both rounds, making it unlikely that large-scale disasters drive our main findings).

Table 1 presents the proportion of households who experienced economic shocks and/or mental health disorders. Overall, 16.3% (N=1155) of individuals experienced depression (PHQ-9 score ≥10), 6.5% (N=459) reported anxiety (GAD-7 score ≥10) and 5.1% experienced both depression and anxiety (N=361). 71% (N=5034) of households reported at least one economic shock in each round. However, among those, 32.9% were shocks without losses and 38.1% involved some type of loss. Considering only shocks that resulted in losses, 34.4% (N=2438) were income losses, 19.8% (N=1402) asset losses and 21.5% (N=1527) production losses. (online supplemental appendix table A3a presents the distribution of income, asset, and production losses by shock type (eg, drought, flood, extreme heat). Among households with any loss, income loss is the most common across most shock types. In terms of correlations, asset and production losses show the weakest association (column 6), while income loss is more strongly correlated with both asset loss (column 4) and production loss (column 5). For most shock types, the proportion of individuals reporting depression and anxiety is significantly higher among those who experienced a shock compared with those who did not. We also find that there is no statistically significant gender difference in mental health outcomes. However, men are significantly more likely to experience shocks, which is consistent with the cultural context in Bangladesh where men are more likely to participate in the labour market and thus face greater exposure to economic shocks. These summary statistics are presented in online supplemental appendix table A3b. Table 2 presents the estimated effects of different kinds of economic shocks on depression and anxiety. Experiencing any shock, regardless of its economic nature, is not significantly associated with depression (0.005, 95% CI −0.031 to 0.041). However, income, asset and production losses are each associated with a significant increase in the likelihood of depression by 6.1 (95% CI 0.029 to 0.093), 6.4 (95% CI 0.026 to 0.103) and 5.2 (95% CI 0.017 to 0.088) percentage points, respectively. While experiencing any shock does not significantly affect anxiety (0.009, 95% CI −0.012 to 0.030), income and asset losses increase the likelihood of anxiety by 3.9 (95% CI 0.016 to 0.062) and 3.4 (95% CI 0.009 to 0.058) percentage points, respectively. Production losses do not have a statistically significant effect (0.017, 95% CI −0.007 to 0.041) (table 2).

**Table 1:** Proportion of observations affected by mental health and shock

Proportion of mental health and shock variables across both rounds		
Variables:	Percentage	N
Depressed	16.30%	1155
Anxiety	6.50%	459
Both depression and anxiety	5.10%	361
Any shock	71.00%	5034
Shocks with no losses	32.90%	2330
Shocks with losses	38.10%	2704
Break-down of shocks with losses:		
Income loss	34.40%	2438
Asset loss	19.80%	1402
Production loss	21.50%	1527

Proportion of mental health outcomes by shock types						
	Depressed			Anxiety		
	Percentage	N	P value (shock/loss vs none)	Percentage	N	P value (shock/loss vs none)
Any shock	16.80%	845	0.077	6.90%	347	0.024
No shock	15.10%	310		5.50%	112	
Income loss	20.80%	508	<0.0001	9.30%	227	<0.0001
No income loss	13.90%	647		5.00%	232	
Asset loss	22.70%	318	<0.0001	9.30%	131	<0.0001
No asset loss	14.70%	837		5.80%	328	
Production loss	19.90%	304	<0.0001	6.80%	104	0.546
No production loss	15.30%	851		6.40%	355	

**Table 2:** Impact of different shocks on depression and anxiety

	Coefficient (95% CI)	P value	Coefficient (95% CI)	P value
Dependent variable:	Likelihood of depression		Likelihood of depression	
Any kind of shock	0.005 (−0.031 to 0.041)	0.778		
Income loss			0.061 (0.029 to 0.093)	<0.0001
Survey round dummy	0.034 (0.008 to 0.060)	0.010	0.036 (0.013 to 0.059)	0.0020
Wealth	−0.005 (−0.017 to 0.007)	0.41	−0.005 (−0.017 to 0.007)	0.41
No. of observations	7090		7090	
No. of households	3610		3610	
Dependent variable:	Likelihood of depression		Likelihood of depression	
Asset loss	0.064 (0.026 to 0.103)	0.0010		
Production loss			0.052 (0.017 to 0.088)	0.0040
Survey round dummy	0.031 (0.008 to 0.053)	0.010	0.031 (0.008 to 0.054)	0.010
Wealth	−0.005 (−0.017 to 0.007)	0.44	−0.006 (−0.018 to 0.007)	0.37
No. of observations	7090		7090	
No. of households	3610		3610	
Dependent variable:	Likelihood of anxiety		Likelihood of anxiety	
Any kind of shock	0.009 (−0.012 to 0.030)	0.40		
Income loss			0.039 (0.016 to 0.062)	0.0010
Survey round dummy	−0.007 (−0.025 to 0.010)	0.41	−0.007 (−0.023 to 0.009)	0.38
Wealth	0.001 (−0.007 to 0.010)	0.804	0.001 (−0.007 to 0.010)	0.77
No. of observations	7090		7090	
No. of households	3610		3610	
Dependent variable:	Likelihood of anxiety		Likelihood of anxiety	
Asset loss	0.034 (0.009, 0.058)	0.010		
Production loss			0.017 (−0.007 to 0.041)	0.16
Survey round dummy	−0.010 (−0.026 to 0.006)	0.21	−0.009 (−0.026 to 0.007)	0.24
Wealth	0.001 (−0.007 to 0.010)	0.74	0.001 (−0.007 to 0.010)	0.80
No. of observations	7090		7090	
No. of households	3610		3610	

In addition to examining depression and anxiety separately, we also assess their co-occurrence and the cumulative effects of experiencing multiple types of shocks (eg, droughts, flood, extreme heat) on mental health (table 3). (Here, ‘number of shocks’ refers to the count of different types of shocks experienced. It does not count the number of specific losses (eg, income, asset or production losses) resulting from a shock, nor does it capture repeated incidents of the same shock type within the six-month recall period, as the survey recorded only the initial occurrence in the last 6 months and the duration of each shock. Consistent with previous results, income losses (0.033, 95% CI 0.013 to 0.053) and asset losses (0.030, 95% CI 0.007 to 0.053) are significantly associated with an increased likelihood of experiencing depression and anxiety simultaneously, while production losses have no significant effect. The results also show that experiencing a greater number of shock types is associated with greater mental health problems. Experiencing a single type of economic shock is significantly associated with a 4.7 percentage-point increase in the likelihood of depression (95% CI 0.015, 0.080), while experiencing two or more types nearly doubles this effect to 9.1 percentage points (95% CI 0.046, 0.136). A similar doubling of effects is observed for anxiety and the co-occurrence of depression and anxiety (table 3).

**Table 3:** Impact of shocks on both depression and anxiety, the impact of number of shocks, and the influence of coping mechanisms

	Coefficient (95% CI)	P value	Coefficient (95% CI)	P value	Coefficient (95% CI)	P value
Dependent variable:	Both depression and anxiety		Both depression and anxiety		Both depression and anxiety	
Income loss	0.033 (0.013 to 0.053)	0.0010				
Asset loss			0.030 (0.007 to 0.053)	0.011		
Production loss					0.012 (−0.007 to 0.031)	0.223
Dependent variable:	Depression		Anxiety		Both depression and anxiety	
Experienced one shock type	0.047 (0.015 to 0.080)	0.010	0.02 (−0.001 to 0.042)	0.058	0.015 (−0.003 to 0.032)	0.107
Experienced two or more shock types	0.091 (0.046 to 0.136)	<0.0001	0.054 (0.019 to 0.089)	0.0030	0.046 (0.014 to 0.078)	0.005
Dependent variable	Depression		Depression		Depression	
Income loss without coping mech.	0.065 (0.030 to 0.101)	<0.0001				
Asset loss without coping mech.			0.059 (0.015 to 0.103)	0.009		
Production loss without coping mech.					0.062 (0.021 to 0.103)	0.003
Dependent variable	Depression		Depression		Depression	
Income loss with coping mech.	0.004 (−0.052 to 0.060)	0.896				
Asset loss with coping mech.			0.051 (−0.028 to 0.129)	0.206		
Production loss with coping mech.					−0.007 (−0.065 to 0.052)	0.822
Dependent variable	Anxiety		Anxiety		Anxiety	
Income loss without coping mech.	0.028 (0.007 to 0.049)	0.008				
Asset loss without coping mech.			0.025 (−0.003 to 0.053)	0.081		
Production loss without coping mech.					0.021 (−0.003 to 0.045)	0.087
Dependent variable	Anxiety		Anxiety		Anxiety	
Income loss with coping mech.	0.034 (−0.012 to 0.079)	0.148				
Asset loss with coping mech.			0.047 (−0.011 to 0.105)	0.115		
Production loss with coping mech.					−0.004 (−0.039 to 0.030)	0.802

Note: The sample size for all estimations is 7090 and the number of households are 3610.

The survey also asks households experiencing shocks how they coped with the shocks. Overall, 48.7% of households reporting any shock used a coping mechanism, with substantially higher rates among those reporting losses. Across income, asset and production losses, 78%–81.9% of households adopted some coping strategy. Savings and credit were the most common, followed by assistance from relatives or friends, increased non-farm work and migration. Patterns were broadly similar across all three types of shocks. We provide these results in online supplemental appendix table A4, for reference.

As coping mechanisms following shocks may influence mental health, we analysed the impact of shocks separately for individuals who used a coping mechanism and those who did not. As shown in table 3, individuals who experienced losses and did not use any coping mechanism were significantly more likely to report depression and anxiety. For example, income, asset and production losses without coping were associated with statistically significant increases in the likelihood of depression by 6.5 percentage points (95% CI 0.030, 0.101), 5.9 percentage points (95% CI 0.015, 0.103) and 6.2 percentage points (95% CI 0.021, 0.103), respectively. These estimates are similar to the main results in table 2. By contrast, individuals who used a coping mechanism did not exhibit increased symptoms following economic losses. Although potential reverse causality cannot be ruled out, as mental health may affect the capacity to adopt coping strategies, the consistency and direction of the results suggest that coping mechanisms may play a protective role in moderating the mental health impacts of economic shocks.

We next examine whether education buffers the adverse mental health effects of economic shocks. table 4 presents the results from an interaction model incorporating a dummy variable for completion of primary education. (Because adult education does not change over the 6-month interval between survey rounds, the education dummy is time-invariant and therefore drops out of the individual fixed effects estimations). The interaction terms are negative and statistically significant for depression for all three types of shocks, suggesting that individuals with at least a primary education are less vulnerable to depression following economic shocks. A weaker buffering effect is observed for anxiety, with only the interaction term for asset loss being statistically significant. Shocks are significantly associated with increases in both the depression and anxiety indices.

**Table 4:** Buffering effects of primary education on mental health in response to shocks

	Coefficient (95% CI)	P value	Coefficient (95% CI)	P value	Coefficient (95% CI)	P value
Dependent variable: Likelihood of depression						
Completed Primary x						
Income loss	−0.047 (−0.095 to 0.001)	0.057				
Asset loss			−0.090 (−0.154 to –0.027)	0.0050		
Production loss					−0.106 (−0.167 to −0.045)	0.0010
Income loss	0.085 (0.043 to 0.127)	<0.0001				
Asset loss			0.111 (0.053 to 0.170)	<0.0001		
Production loss					0.110 (0.055 to 0.164)	<0.0001
Number of observations	7090		7090		7090	
Number of households	3610		3610		3610	
Dependent variable: Likelihood of anxiety						
Completed Primary x						
Income loss	−0.016 (−0.057 to 0.024)	0.42				
Asset loss			−0.049 (−0.090 to –0.007)	0.022		
Production loss					−0.025 (−0.065 to –0.015)	0.22
Income loss	0.048 (0.017 to 0.078)	0.0020				
Asset loss			0.059 (0.024 to 0.095)	0.0010		
Production loss					0.031 (−0.005 to 0.067)	0.095
Number of observations	7090		7090		7090	
Number of households	3610		3610		3610	

Given the potential protective role of education through coping mechanisms, online supplemental appendix table A5 compares coping responses by education level. Individuals without primary education were more likely to report using different coping mechanisms than those with primary education across most shocks. The exception was reliance on savings, which was more common among those with primary education across all loss types.

To explore potential pathways linking shocks to mental health, we examined indicators of physical health and food security. Respondents were asked whether they experienced injury or illness in the prior 30 days and, if so, whether they sought medical assistance, and those reporting shocks were also asked if their food purchases declined. As shown in online supplemental table A6, shocks causing asset or production losses were associated with a significantly higher likelihood of injury or illness, and income and asset losses were associated with a higher likelihood of seeking medical assistance. In addition, a substantial proportion of households reporting losses also reported a decline in food purchases: 54.2% for income loss, 61.8% for asset loss and 75.8% for production loss (online supplemental table A6).

We assess the medium-run effects of economic shocks by examining whether shocks in round 1 predict mental health outcomes 6–12 months later in round 2 (table 5). Given the cross-sectional nature of the data in this analysis, individual fixed effects cannot be applied. Instead, the models control for round 2 shocks, wealth and individual characteristics including gender, age, relation to household head, marital status, primary education completion, district fixed effects and urban or rural residence. Given the absence of individual fixed effects and potential multicollinearity between rounds 1 and 2 shocks, these results should be interpreted with caution.

**Table 5:** Impact of shocks in round 1 on depression and anxiety in the following round

	Coefficient (95% CI)	P value	Coefficient (95% CI)	P value	Coefficient (95% CI)	P value
Dependent variable: Likelihood of depression in round 2						
Income loss in round 1	0.064 (0.022 to 0.107)	0.003				
Income loss in round 2	−0.014 (−0.020 to –0.007)	0.000				
Asset loss in round 1			0.041 (−0.004 to 0.085)	0.072		
Asset loss in round 2			0.116 (0.070 to 0.161)	0.000		
Production loss in round 1					0.034 (−0.006 to 0.074)	0.091
Production loss in round 2					0.036 (0.002 to 0.070)	0.039
Number of observations	3480		3480		3480	
Dependent variable: Likelihood of anxiety in round 2						
Income loss in round 1	0.016 (−0.002 to 0.034)	0.084				
Income loss in round 2	0.040 (0.006 to 0.073)	0.022				
Asset loss in round 1			−0.001 (−0.026 to 0.025)	0.948		
Asset loss in round 2			0.042 (0.007 to 0.078)	0.018		
Production loss in round 1					0.005 (−0.020 to 0.030)	0.705
Production loss in round 2					−0.013 (−0.035 to 0.009)	0.256
Number of observations	3480		3480		3480	

The results indicate that income loss (0.064, 95% CI 0.022 to 0.107), asset loss (0.041, 95% CI −0.004 to 0.085) and production loss (0.034, 95% CI −0.006 to 0.074) in round 1 are all significantly associated with higher likelihood of depression in round 2. For anxiety, only income loss has a statistically significant effect (0.016, 95% CI −0.002 to 0.034).

### Sensitivity analyses

We conducted several sensitivity analyses for our estimations. To address potential measurement error in the PHQ-9 and GAD-7 scales, we conduct two robustness checks.

First, one concern is that relying on specific cut-off points in the PHQ-9 and GAD-7 scales may overlook potential nonlinearities in the measurement of depression and anxiety. To address this issue, we standardise both scores to continuous indices ranging from 0 to 1. We find that the shocks were significantly associated with higher levels of both depression and anxiety.

Second, the cut-off of 10 for both scales may be too low and could overestimate depression and anxiety. A score of 10 on the PHQ-9 indicates moderate depression or higher. We applied a stricter threshold of 15—indicating moderate-to-severe symptoms—to define binary indicators of ‘high depression’ and ‘high anxiety’. The results remained robust, with shocks significantly increasing the likelihood of both outcomes. Both of these results are in online supplemental appendix table A7.

Beyond high mental distress, we analysed whether shocks are linked to moderate and mild depression and anxiety. We created indicators for moderate depression/anxiety (PHQ-9 and GAD-7 scores 10–14) and mild depression/anxiety (scores 5–9). Results are presented in online supplemental appendix table A8. Production loss was associated with both mild and moderate depression, while income loss was associated with moderate depression only. For anxiety, income loss was associated with both mild and moderate cases, whereas production loss was associated with moderate anxiety. In contrast, asset loss showed no significant association with mild or moderate depression or anxiety.

We also examined the potential for reverse causality, as long-term depression may lead individuals to experience or report more shocks. To mitigate this, we excluded individuals who were depressed in both rounds, thus isolating those with more transient symptoms. The results remained robust, indicating that the observed associations are not driven by long-term depression. The results are presented in online supplemental appendix table A9.

To further assess the potential for reverse causality, we tested whether mental health issues in the first round influenced the likelihood of reporting shocks in the second round. We find that anxiety in round 1 did not significantly correlate with the reporting of shocks in round 2. Although depression in round 1 was not associated with income or asset losses, it did significantly reduce the likelihood of reporting a production loss in round 2. The results are presented in online supplemental appendix table A10.

A further concern is that individual-level shocks may be subject to measurement errors, endogeneity and reverse causality, such as individuals with better mental health may be more able to mitigate shocks. We address such concerns with individual-level data by aggregating shocks to the subdistrict and district levels and regressing these aggregates on mental health measures. At the subdistrict level, income and production losses significantly increase the likelihood of depression and anxiety, while asset loss has no significant effect. (SEs are clustered at the subdistrict level for subdistrict-level aggregate shocks and at the district level for district-level shocks). At the district level, all three types of shocks are significantly associated with both depression and anxiety. Both sets of results are presented in online supplemental appendix table A11.

## Discussion

This study provides evidence associating economic shocks with adverse mental health outcomes in an LMIC setting. Our findings suggest that economic shocks, particularly those involving income and asset loss, significantly increase the likelihood of depression and anxiety. In contrast, the ‘any shock’ measure shows no significant association with mental health. This difference reflects how the variable is defined: ‘any shock’ includes both shocks that resulted in economic losses (income, asset or production) and shocks that did not lead to losses. Because the latter are generally less severe and unlikely to affect mental health, the broader ‘any shock’ measure masks the effects of loss-related shocks, whereas specific indicators of income, asset or production losses more clearly capture the disruptions that drive mental health outcomes.

There is evidence of medium-run effects. Shocks in Round 1 are associated with mental health outcomes in round 2, indicating that impacts remain evident for at least six to twelve months after the initial shock. With the exception of the association between income loss and depression, the coefficients for round 1 shocks (table 5) are smaller than the short-term estimates reported for the full sample in table 2. This suggests that although the effects generally persist, their magnitudes tend to gradually weaken over time.

Importantly, the cumulative exposure to different types of economic shocks exacerbates psychological distress, highlighting the compounding nature of economic adversity. Our study provides support to the social causation theory of mental illness, where socioeconomic hardships can lead to mental illness.^38^ The strong association between shocks and mental health outcomes aligns with previous evidence that financial insecurity is a key driver of psychological distress.^39 40^ Loss of income may heighten financial and psychological strain, limit access to essential resources and increase uncertainty, all of which contribute to worsened mental health. Similarly, asset depletion may signal long-term economic hardship, further intensifying stress and anxiety. However, we could not identify support for the social drift hypothesis,^38^ which suggests an increased vulnerability to future economic shocks among those with a mental health condition. This does not necessarily invalidate the presence of other social drift mechanisms but provides empirical evidence that suggests economic shocks as a proxy for social drift may not have utility in Bangladesh’s context.

The weaker effects of production losses may reflect the nature of household economic arrangements. Unlike income or asset loss, food production disruptions may not immediately translate into financial hardship if households have alternative income sources or other buffer mechanisms. However, the cumulative burden of multiple types of shocks substantially worsens mental health outcomes, reinforcing the idea that resilience to economic stress diminishes with repeated exposure.

We find that income loss and production loss are associated with varying levels of depression and anxiety, spanning mild, moderate and more severe cases. In contrast, asset loss is distinct in its effects: it is significantly associated only with severe depression and anxiety, but not with mild or moderate cases. These findings indicate that asset losses primarily contribute to severe mental health outcomes, as opposed to less intense forms of distress. Our findings suggest several pathways through which economic shocks may affect mental health. The absence of effective coping mechanisms appears central, as individuals without access to such strategies were primarily affected by depression and anxiety following losses, whereas those able to use coping mechanisms did not experience significant adverse mental health effects. We also observe associations between shocks and recent physical illness or injury, as well as reduced food purchases, the latter indicating higher food insecurity among affected households. These channels may interact to exacerbate psychological distress. Taken together, our results suggest that the mental health effects of shocks likely operate through several interrelated mechanisms, highlighting the need for interventions that both strengthen coping capacity and address basic needs such as health and food security during periods of economic disruption.

Individuals with at least a primary education exhibited lower vulnerability to depression following economic shocks, though this buffering effect was less pronounced for anxiety. These findings on education as a protective factor align with broader literature on education having a positive impact on mental health in the context of LMIC.^41^,^44^ When examining differences in coping mechanisms by education, individuals who completed primary schooling were more likely to be able to rely on savings than those who did not. This pattern can suggest that education may enable individuals to earn higher incomes and accumulate resources, increasing their ability to save and, in turn, providing a protective buffer against adverse mental health outcomes. These results underscore the potential of education as a long-term intervention to mitigate the mental health impact of economic fluctuations.

This study benefits from a longitudinal design with individual fixed effects, allowing for robust identification of causal relationships between economic shocks and mental health outcomes. The use of multiple robustness checks, including alternative measurement approaches, strengthens the validity of our findings. However, some limitations must be acknowledged. First, self-reported measures of economic shocks may be subject to recall or reporting biases. Second, we are unable to use a clinician’s medical diagnosis of depression and anxiety and instead rely on self-reported data. Third, while our analysis of medium-run effects suggests persistence in mental health impacts, the study’s two-wave structure limits our ability to assess long-term consequences. Future research with longer follow-up periods is needed to understand the prolonged impact of economic instability on mental health.

Our findings have important policy implications in the context of countries in similar resource-poor settings. Given the strong association between financial hardship and mental health outcomes, interventions aimed at improving economic security, such as income support programmes or employment protections during major shocks, may serve as effective strategies to mitigate psychological distress, which has been shown in prior studies.^45^,^47^

Additionally, expanding mental health services, particularly for populations experiencing economic instability, could help reduce the burden of depression and anxiety. Furthermore, the protective role of education suggests that investments in human capital could yield long-term mental health benefits. Policies aimed at increasing access to quality education may not only improve economic opportunities but also enhance resilience to financial shocks. Overall, it is essential to implement comprehensive strategies that bolster financial stability, strengthen social safety nets and improve mental health support in low-income contexts.

## Supplementary material

10.1136/bmjgh-2025-020502 online supplemental file 1

## Data Availability

Data are available on reasonable request.
