# Supplementary material for: Economic shocks and mental health in Bangladesh
Source: BMJ Glob Health. 2025 Dec 25;10(12):e020502. doi: 10.1136/bmjgh-2025-020502 (PMC12742073; doi:10.1136/bmjgh-2025-020502)
Supplement: online supplemental file 1 [file bmjgh-10-12-s001.pdf]

Appendix

Table A1: Summary statistics of respondent characteristics

	Overall sample	Individuals who experienced shocks that caused losses	Individuals who did not experience loss-causing shocks	Difference between columns 2 and 3	P-value of the differences between columns 2 and 3
	(1)	(2)	(3)	(4)	(5)
Male	40.9%	47.6%	36.8%	10.8%	<0·0001
Female	59.1%	52.4%	63.2%	-10.8%	<0·0001
Currently Married	86.0%	87.5%	85.1%	2.4%	0.005
Never Married	6.21%	5.77%	6.48%	-0.7%	0.231
Widowed, divorced or separated	7.79%	6.77%	8.41%	-1.6%	0.012
Age	39.9	40.4	39.6	0.80	0.025
Completed primary education or higher	56.60%	48.37%	61.67%	-13.3%	<0·0001
Wealth measure (prin. component)	0.109	0.091	0.120	-0.03	<0·0001
Rural	60.6%	74.5%	52.1%	22.4%	<0·0001
Urban	39.4%	25.5%	47.9%	-22.4%	<0·0001

Table A2: Fraction of households reporting different shock types and losses across two rounds

	First survey round				Second survey round				Fraction of households experienced the shock in both rounds
	Any reporting of the particular shock	Shock led to income loss	Shock led to asset loss	Shock led to production loss	Any reporting of the particular shock	Shock led to income loss	Shock led to asset loss	Shock led to production loss	
Drought or irregular rainfall	33.5%	12.6%	5.2%	5.4%	18.2%	7.3%	3.8%	3.8%	5.2%
Floods	27.0%	14.2%	6.1%	7.9%	20.2%	12.0%	8.3%	9.1%	6.7%
Extreme heat events	60.6%	16.9%	4.6%	4.7%	27.4%	6.2%	1.5%	2.5%	12.8%
Landslides or erosion	1.9%	1.2%	1.1%	1.1%	0.6%	0.5%	0.4%	0.2%	0.2%
Crop pest	4.1%	2.8%	1.2%	2.2%	10.5%	7.4%	4.7%	6.5%	0.5%
Livestock disease	6.0%	3.5%	3.0%	1.5%	15.7%	7.2%	6.5%	4.3%	1.1%
Increase in agricultural input prices	11.0%	4.6%	2.6%	4.7%	7.6%	2.5%	1.1%	2.3%	0.3%
Decrease in agricultural output prices	19.2%	11.6%	5.3%	7.2%	9.8%	7.1%	3.2%	3.9%	2.2%
Natural disasters	1.1%	0.1%	0.2%	0.1%	5.7%	1.4%	1.8%	1.0%	0.0%

Table A3a: Percentage of households experiencing losses among households that experienced at least an income, asset, or production loss

	Income loss	Asset loss	Production loss	Both income and asset loss	Both income and production loss	Both asset and production loss	Loss of income, asset, and production
	(1)	(2)	(3)	(4)	(5)	(6)	(7)
Drought or irregular rainfall	95.7%	42.9%	44.1%	40.2%	40.8%	32.0%	30.2%
Floods	95.7%	52.8%	61.9%	50.0%	58.9%	45.6%	44.1%
Extreme heat events	97.3%	25.4%	29.9%	23.7%	27.8%	20.7%	19.6%
Landslides or erosion	95.2%	90.3%	77.4%	85.5%	74.2%	69.4%	66.1%
Crop pest	84.5%	48.9%	71.8%	38.6%	57.4%	35.5%	26.4%
Livestock disease	73.5%	65.5%	39.6%	41.2%	32.5%	28.4%	23.5%
Increase in agricultural input prices	71.7%	37.4%	70.0%	30.9%	44.8%	25.5%	22.1%
Decrease in agricultural output price	97.1%	44.0%	57.4%	43.0%	55.0%	28.1%	27.6%
Natural disasters	55.0%	67.0%	37.0%	27.0%	27.0%	27.0%	22.0%

Table A3b: Difference in mental health and shocks by gender

	Male	Female	Difference between male and female	P-value of difference between male and female
Depressed	15.7%	16.7%	-1.0%	0.273
Anxiety	6.9%	6.2%	0.8%	0.197
Both depression and anxiety	5.6%	4.8%	0.8%	0.117
Any shock	74.5%	68.6%	5.9%	<0·0001
Income loss	40.3%	30.3%	10.0%	<0·0001
Asset loss	23.2%	17.4%	5.8%	<0·0001
Production loss	26.6%	18.0%	8.6%	<0·0001

Table A4: Different coping mechanisms following shocks

	Fraction of individuals who experienced any shock and used the coping measure	Fraction of individuals who experienced income loss and used the coping measure	Fraction of individuals who experienced asset loss and used the coping measure	Fraction of individuals who experienced production loss and used the coping measure	Fraction of overall sample that used the coping measure
Any coping measure taken following a shock	48.7%	78.0%	81.6%	81.9%	35.7%
Major coping measures:					
Relied on savings	31.6%	48.9%	53.5%	50.6%	23.0%
Obtained credit	27.3%	50.7%	55.6%	54.8%	19.9%
Help provided by relatives/friends	8.0%	14.7%	14.4%	18.1%	6.0%
Household member took on more non-farm work	2.1%	3.9%	3.9%	5.5%	1.5%
Household members migrated	1.5%	2.8%	3.4%	4.4%	1.1%

Table A5: Coping mechanisms following shocks by different education levels

	Fraction of individuals who experienced any shock and used the coping measure		Fraction of individuals who experienced income loss and used the coping measure	
	Finished primary school	Did not finish primary school	Finished primary school	Did not finish primary school
Any coping measure taken following a shock	44.1%	54.4%	77.8%	78.2%
Major coping measures:				
Relied on savings	31.1%	32.3%	53.0%	45.3%
Obtained credit	22.0%	33.7%	46.2%	54.7%
Help provided by relatives/friends	5.6%	11.0%	12.6%	16.6%
Household member took on more non-farm work	2.0%	2.2%	4.2%	3.6%
Household members migrated	1.3%	1.8%	2.9%	2.7%

	Fraction of individuals who experienced asset loss and used the coping measure		Fraction of individuals who experienced production loss and used the coping measure	
	Finished primary school	Did not finish primary school	Finished primary school	Did not finish primary school
Any coping measure taken following a shock	79.3%	83.8%	79.4%	84.3%
Major coping measures:				
Relied on savings	55.5%	51.5%	53.0%	48.3%
Obtained credit	49.6%	61.6%	49.4%	60.0%
Help provided by relatives/friends	11.8%	17.0%	14.7%	21.5%
Household member took on more non-farm work	3.7%	4.0%	5.7%	5.3%
Household members migrated	3.3%	3.6%	4.3%	4.5%

Table A6: Association between shocks and injury or illness, seeking of medical assistance, and food security

	Coefficient (95% CI)	P-value	Coefficient (95% CI)	P-value	Coefficient (95% CI)	P-value
Dependent variable: Likelihood of illness or injury in the last 30 days						
Income loss	0.028 (-0.008 - 0.065)	0.121				
Asset loss			0.060 (0.019 - 0.100)	0.004		
Production loss					0.046 (0.002 - 0.090)	0.043
Number of observations	7,090		7,090		7,090	
Number of households	3,610		3,610		3,610	
Dependent variable: Likelihood of seeking medical assistance following illness						
Income loss	0.073 (-0.000 - 0.146)	0.051				
Asset loss			0.094 (0.002 - 0.186)	0.045		
Production loss					0.051 (-0.040 - 0.143)	0.269
Number of observations	2,617		2,617		2,617	
Number of households	2,006		2,006		2,006	
Fraction of individuals who experienced losses who also experienced a decline in food purchase						
Income loss	54.2%					
Asset loss	61.8%					
Production loss	75.8%					

Table A7: Impact of shocks on mental health indices and on high depression and anxiety

	Coefficient (95% CI)	P-value	Coefficient (95% CI)	P-value	Coefficient (95% CI)	P-value
Dependent variable: Depression index						
Income loss	0.036 (0.020, 0.053)	<0·0001				
Asset loss			0.038 (0.017, 0.059)	0.0010		
Production loss					0.040 (0.021, 0.059)	<0·0001
Dependent variable: Anxiety index						
Income loss	0.039 (0.021, 0.057)	<0·0001				
Asset loss			0.025 (0.004, 0.046)	0.019		
Production loss					0.037 (0.017, 0.056)	<0·0001
Dependent variable: Likelihood of high depression						
Income loss	0.028 (0.007, 0.050)	0.010				
Asset loss			0.041 (0.012, 0.069)	0.010		
Production loss					0.024 (0.001, 0.048)	0.039
Dependent variable: Likelihood of high depression						
Income loss	0.012 (0.000, 0.024)	0.049				
Asset loss			0.018 (0.001, 0.036)	0.042		
Production loss					-0.000 (-0.012, 0.011)	0.98
Number of observations	7,090		7,090		7,090	
Number of households	3,610		3,610		3,610	

Table A8: Impact of shocks on mild and moderate mental health issues

	Coefficient (95% CI)	P-value	Coefficient (95% CI)	P-value	Coefficient (95% CI)	P-value
Dependent variable: Mild depression						
Income loss	0.007 (-0.034, 0.048)	0.732				
Asset loss			-0.010 (-0.058, 0.037)	0.673		
Production loss					0.055 (0.014, 0.095)	0.008
Dependent variable: Moderate depression						
Income loss	0.033 (0.006, 0.059)	0.017				
Asset loss			0.024 (-0.007, 0.054)	0.123		
Production loss					0.028 (-0.000, 0.056)	0.052
Dependent variable: Mild anxiety						
Income loss	0.046 (0.002, 0.091)	0.041				
Asset loss			-0.004 (-0.050, 0.043)	0.869		
Production loss					0.074 (0.026, 0.121)	0.003
Dependent variable: Moderate anxiety						
Income loss	0.027 (0.009, 0.046)	0.004				
Asset loss			0.015 (-0.003, 0.034)	0.107		
Production loss					0.017 (-0.005, 0.039)	0.121
Number of observations	7,090		7,090		7,090	
Number of households	3,610		3,610		3,610	

Table A9: Impact on mental health where individuals with depression or anxiety in both rounds are dropped

	Coefficient (95% CI)	P-value	Coefficient (95% CI)	P-value	Coefficient (95% CI)	P-value
Dependent variable: Likelihood of depression						
Income loss	0.063 (0.030, 0.097)	<0·0001				
Asset loss			0.068 (0.027, 0.110)	0.0010		
Production loss					0.055 (0.017, 0.092)	0.0040
Number of observations	6,726		6,726		6,726	
Number of households	3,428		3,428		3,428	

Dependent variable: Likelihood of anxiety						
Income loss	0.040 (0.016, 0.063)	0.0010				
Asset loss			0.034 (0.009, 0.059)	0.010		
Production loss					0.017 (-0.007, 0.042)	0.16
Number of observations	7,006		7,006		7,006	
Number of households	3,568		3,568		3,568	

Table A10: Impact of mental health problem in round 1 on reporting of shocks in round 2

	Coefficient (95% CI)	P-value	Coefficient (95% CI)	P-value	Coefficient (95% CI)	P-value
Dependent variable:	Income loss in round 2		Asset loss in round 2		Production loss in round 2	
Depression in round 1	-0.038 (-0.091, 0.015)	0.16	-0.018 (-0.065, 0.028)	0.44	-0.047 (-0.095, 0.002)	0.059
No of observations	3,480		3,480		3,480	

Dependent variable:	Income loss in round 2		Asset loss in round 2		Production loss in round 2	
Anxiety in round 1	0.014 (-0.067, 0.094)	0.74	0.018 (-0.064, 0.100)	0.67	-0.026 (-0.096, 0.044)	0.46
No of observations	3,480		3,480		3,480	

Table A11: Impact of aggregate regional shocks on depression and anxiety

	Coefficient (95% CI)	P-value	Coefficient (95% CI)	P-value	Coefficient (95% CI)	P-value	Coefficient (95% CI)	P-value
Shocks aggregated at the sub-district level:								
Dependent variable: Likelihood of depression								
Any kind of shock	-0.036 (-0.153, 0.081)	0.538						
Income loss			0.221 (0.097, 0.345)	0.001				
Asset loss					0.105 (0.097, 0.345)	0.128		
Production loss							0.175 (0.041, 0.308)	0.011
Dependent variable: Likelihood of anxiety								
Any kind of shock	-0.005 (-0.077, 0.068)	0.897						
Income loss			0.144 (0.065, 0.223)	0.001				
Asset loss					0.045 (-0.050, 0.139)	0.350		
Production loss							0.134 (0.031, 0.237)	0.012
Shocks aggregated at the district level:								
Dependent variable: Likelihood of depression								
Any kind of shock	-0.071 (-0.225, 0.083)	0.356						
Income loss			0.325 (0.171, 0.479)	<0·0001				
Asset loss					0.304 (0.091, 0.517)	0.006		
Production loss							0.287 (0.119, 0.455)	0.001
Dependent variable: Likelihood of anxiety								
Any kind of shock	0.025 (-0.070, 0.120)	0.597						
Income loss			0.162 (0.068, 0.256)					
Asset loss					0.156 (0.052, 0.260)	0.004		
Production loss							0.150 (0.041, 0.259)	0.008
No. of observations	7,090		7,090		7,090		7,090	
No. of households	3,610		3,610		3,610		3,610	
No. of sub-districts	65		65		65		65	
No. of districts	53		53		53		53	
